# Mnemonic But Not Contextual Feedback Signals Defy Dedifferentiation in the Aging Early Visual Cortex

**DOI:** 10.1523/JNEUROSCI.0607-23.2023

**Published:** 2024-02-23

**Authors:** Isabelle Ehrlich, Javier Ortiz-Tudela, Yi You Tan, Lars Muckli, Yee Lee Shing

**Affiliations:** ^1^Department of Psychology, Goethe University Frankfurt, Frankfurt Am Main 60323, Germany; ^2^Department of Experimental Psychology, Mind, Brain, and Behavior Research Center, University of Granada, Granada 18013, Spain; ^3^School of Psychology and of Neuroscience, University of Glasgow, Glasgow G12 8QB, United Kingdom; ^4^IDeA Center for Individual Development and Adaptive Education, Frankfurt am Main 60323, Germany; ^5^Brain Imaging Center, Goethe University Frankfurt, Frankfurt am Main 60528, Germany

**Keywords:** aging, dedifferentiation, feedback, memory, predictive processing, visual cortex

## Abstract

Perception is an intricate interplay between feedforward visual input and internally generated feedback signals that comprise concurrent contextual and time-distant mnemonic (episodic and semantic) information. Yet, an unresolved question is how the composition of feedback signals changes across the lifespan and to what extent feedback signals undergo age-related dedifferentiation, that is, a decline in neural specificity. Previous research on this topic has focused on feedforward perceptual representation and episodic memory reinstatement, suggesting reduced fidelity of neural representations at the item and category levels. In this fMRI study, we combined an occlusion paradigm that filters feedforward input to the visual cortex and multivariate analysis techniques to investigate the information content in cortical feedback, focusing on age-related differences in its composition. We further asked to what extent differentiation in feedback signals (in the occluded region) is correlated to differentiation in feedforward signals. Comparing younger (18–30 years) and older female and male adults (65–75 years), we found that contextual but not mnemonic feedback was prone to age-related dedifferentiation. Semantic feedback signals were even better differentiated in older adults, highlighting the growing importance of generalized knowledge across ages. We also found that differentiation in feedforward signals was correlated with differentiation in episodic but not semantic feedback signals. Our results provide evidence for age-related adjustments in the composition of feedback signals and underscore the importance of examining dedifferentiation in aging for both feedforward and feedback processing.

## Significance Statement

Cognitive decline in aging is related to reduced neural specificity (dedifferentiation) in the brain, which has mainly been examined in feedforward processing. Using an occlusion paradigm, we tested whether there is dedifferentiation in contextual and mnemonic feedback signals internally generated in the early visual cortex (EVC) to aid perception. Older adults’ contextual but not mnemonic feedback signals suffered from dedifferentiation, with semantic mnemonic representations being even better differentiated in older age. Neural differentiation between feedforward and episodic feedback signals was positively correlated in both age groups. In sum, these results highlight the growing importance of semantic knowledge across the lifespan and imply that the impact of dedifferentiation on cognition highly depends on the nature of the recruited information.

## Introduction

Throughout the lifespan, our brain undergoes structural and functional changes. A notable pattern emerges within the memory systems as we approach later stages of life: episodic memory tends to decline, while semantic memory is mostly preserved (Shing et al., 2008; Ofen and Shing, 2013; Lalla et al., 2022). At the same time, the notion of dedifferentiation has emerged in the literature as an important factor contributing to age-related functional changes in cognition. Age-related dedifferentiation refers to the finding that neural representations become less distinct with advancing age and, therefore, less representative of the evoking stimulus. Support for this view comes from nonhuman animals (Schmolesky et al., 2000; Yang et al., 2008, 2009) and human neuroimaging studies, which showed that neural selectivity for visual stimuli declines with age (Voss et al., 2008; Zheng et al., 2018; Koen et al., 2019, 2020). The visual system has been widely used for studying neural differentiation (Park et al., 2004; Payer et al., 2006; Carp et al., 2010, 2011a,b). Its hierarchical and retinotopic organization allows the mapping of the visual field onto brain voxels, which enables nuanced control of the feedforward input reaching a given portion of the visual cortex. In this study, we combined nuanced control with multivariate analysis techniques to enable us to test the influence of internal models on the constellation and quality of perceptual representations.

State-of-the-art models of perception recognize the brain's heavy reliance on internal representations of the outside world that are formed early in life and updated throughout the lifespan (Berkes et al., 2011; Larkum, 2013; Shin et al., 2021). The predictive processing framework integrates this influence, postulating that feedback signals travel from higher-level brain areas to the earliest sensory regions (Rao and Ballard, 1999; Friston, 2005; Clark, 2013). Importantly, neural units and long-range connections transmitting internally generated feedback signals are distinct from and proportionally more numerous than pathways transmitting external feedforward visual input (Markov et al., 2014). As a result, feedback signals can traverse the visual hierarchy and powerfully drive disambiguation of the percept at early stages. Thus, exploring to what extent age-related dedifferentiation manifests in these top–down-directed perceptual processing streams can provide key insights into the interplay between mnemonic and perceptual systems.

So far, several studies have investigated age-related dedifferentiation in feedback signals mostly in the form of memory reinstatement (St-Laurent et al., 2011, 2014; Bowman et al., 2019; Deng et al., 2021; Katsumi et al., 2021). The overall finding is that dedifferentiation affects the older brains’ integrity by acting on both feedforward sensory input and internally generated representations of information. A recent study by Ortiz-Tudela et al. (2023) showed that feedback signals carry information of different natures. More concretely, their results showed that concurrent contextual and time-distant mnemonic information coexist as feedback signals in primary and secondary visual cortices V1 and V2. Concurrent contextual information refers to visual input that does not reach a given brain region via feedforward but lateral connections. Time-distant mnemonic information describes content drawn from stored knowledge acquired in the past. They found that mnemonic episodic and semantic components explained different portions of the variance of the multivariate neural pattern of feedback signals. Such compositional complexity of feedback signals has not been considered in studies of age-related dedifferentiation so far.

In this study, we combined an occlusion paradigm with fMRI and multivariate pattern analysis (MVPA) to examine the following: (1) if concurrent contextual and time-distant mnemonic information can be decoded in feedback signals within V1 and V2 of older adults, as it was found in younger adults; (2) if feedback signals in older adults are less differentiated; and (3) how the relationship between feedforward and feedback signals is characterized across age. We hypothesized to find contextual and mnemonic information in older adults’ V1 and V2 feedback signals. Compared with contextual feedback, we expected mnemonic episodic, but not semantic, feedback to be impacted by age-related dedifferentiation, as episodic memory decline is a well-established aging phenomenon. Finally, we predicted a positive relationship between feedforward and feedback signals in older compared with younger adults. To our knowledge, this is the first study that examined the detailed interplay of feedforward and feedback components in a cross-sectional lifespan sample.

## Materials and Methods

### Reanalysis of published data and registration

The current study is an extension of a previous study by Ortiz-Tudela et al. (2023), which reported data from 30 younger participants. We collected additional data from younger adults to match the sample size of older adults. All reported results for younger adults in this study refer to the topped-up sample. We preregistered the study prior to data collection on the Open Science Framework (OSF) platform. The preregistration is available at https://doi.org/10.17605/OSF.IO/X7B6Z, and any deviations from it are indicated in the corresponding sections.

### Participants

We conducted sensitivity and a priori power analyses using G*Power (Faul et al., 2007) and validated the results using WebPower (version 0.6; Zhang and Yuan, 2018). Based on the data of Ortiz-Tudela et al. (2023), we obtained a minimal statistically detectable effect size of *f* = 0.37. The sample size calculation resulted in 80 participants, that is, 40 participants per age group to detect this effect with a power of 0.90 and an α of 0.05. For the younger adults’ sample, we reused 30 participants from the Ortiz-Tudela et al. (2023) study and recruited an additional 15 younger adults between 18 and 30 years of age via advertisements across the campus of the Goethe University Frankfurt. We excluded three participants in the younger adults’ sample due to low training performance (<80%) on Day 1, one due to low retrieval performance (<25%) in the postscan phase on Day 2, three due to technical issues during scanning, one because of excessive movement, and one diagnosed with aphantasia. Additionally, we recruited 46 healthy older adults between 65 and 74 years of age via email advertisements to attendees of the university study program for the third age and via invitation letters to residents within the required age range. We excluded two participants due to low training performance (<80%) on Day 1, one due to low retrieval performance (<25%) in the postscan phase on Day 2, two due to MRI incompatibility, one due to technical issues during scanning, and one due to no-show on Day 2. The final sample included 36 younger adults (23 female, *M* = 24.18 years, SD = 2.54) and 39 older adults (18 female, *M* = 69.28 years, SD = 2.99). Before participation, we screened all participants for MRI compatibility, visual capacity, and state of health via phone. We tested older adults additionally with a phone-compatible version of the Mini-Mental State Examination (Folstein et al., 1975) and invited them only if they correctly responded to 16 out of 18 questions. All participants gave written informed consent as approved by the Department for Psychology ethics committee at Goethe University Frankfurt (Protocol number: 2019-38). For their participation, participants received either course credits (only for psychology students) or monetary compensation (8€/h for behavioral tests and 10€/h for the fMRI session).

### Stimuli and materials

The stimulus set consisted of the same diagram image material as in the study from Ortiz-Tudela et al. (2023) and is available at https://github.com/ortiztud/feedbes. It comprised 16 indoor room images (e.g., bathroom, kitchen, electronic store) and 4 object images (i.e., bathtub, oven, bed, and TV). With this material, we created two sets of object–room category pairs. One set consisted of eight combinations with minimal semantic relation (e.g., “bed” in “bathroom”), providing the stimulus material for the episodic trials. The episodic nature of these combinations is given by the need to create new associative memories binding object and room context, which must be retrieved 24 h later during the fMRI scan. The other set consisted of eight combinations with maximal semantic relation (e.g., “oven” in “kitchen”), providing the stimulus material for the semantic trials. Note that the same four objects were used for episodic and semantic trials, but the rooms were unique for each trial type. This was done to ensure comparability between the retrieved object content for episodic and semantic trials and to equate the difficulty between the pairings as much as possible. Object–room combinations and the assignment of room categories to either episodic or semantic trials were counterbalanced across participants. Thereby, we preserved the required (in)congruent relationships in the respective stimulus sets and ensured that every object would be presented in every possible room and in episodic trials as well as semantic trials across the entire sample. Importantly, the objects were always placed in the lower right corner of the room. Depending on the task, they were either visible or hidden behind a white patch that occluded the respective corner. Previous studies successfully used such an occlusion paradigm to separate feedback signals from feedforward visual input (Smith and Muckli, 2010; Muckli et al., 2015; Morgan et al., 2019). During the learning phase on Day 1, we presented the stimuli on a 60 Hz monitor (resolution, 1,680 × 1,050, full HD) approximately 60 cm from the participant's head. Subjects responded using a standard QWERTY keyboard. In the scanner, participants saw the stimuli on a 60 Hz monitor (resolution, 1,920 × 1,080, full HD) via a coil-mounted mirror with an approximate total distance of 162 cm to the participants’ eye. The size of the stimuli spanned 16.4° × 12.1° of visual angle.

### Procedure

For younger adults, the procedure was identical to the original study (Ortiz-Tudela et al., 2023). All necessary adjustments to ensure that older adults can manage the task as similarly as younger adults are specified in the corresponding sections. The procedure was split into two sessions across 2 consecutive days.

### Day 1

The first session took place in a quiet testing room. Participants started the first session by answering a set of questionnaires, including the Vividness of Visual Imagery Questionnaire (Marks, 1973); the Spot-the-Word test (Baddeley et al., 1993); the Digit Symbol test, which is a subtest of the Wechsler Adult Intelligence Scale (Wechsler, 1981); and the Health Dynamics Inventory (Saunders and Wojcik, 2004). After that, they proceeded with the learning phase.

#### Learning phase

**Figure 1.**
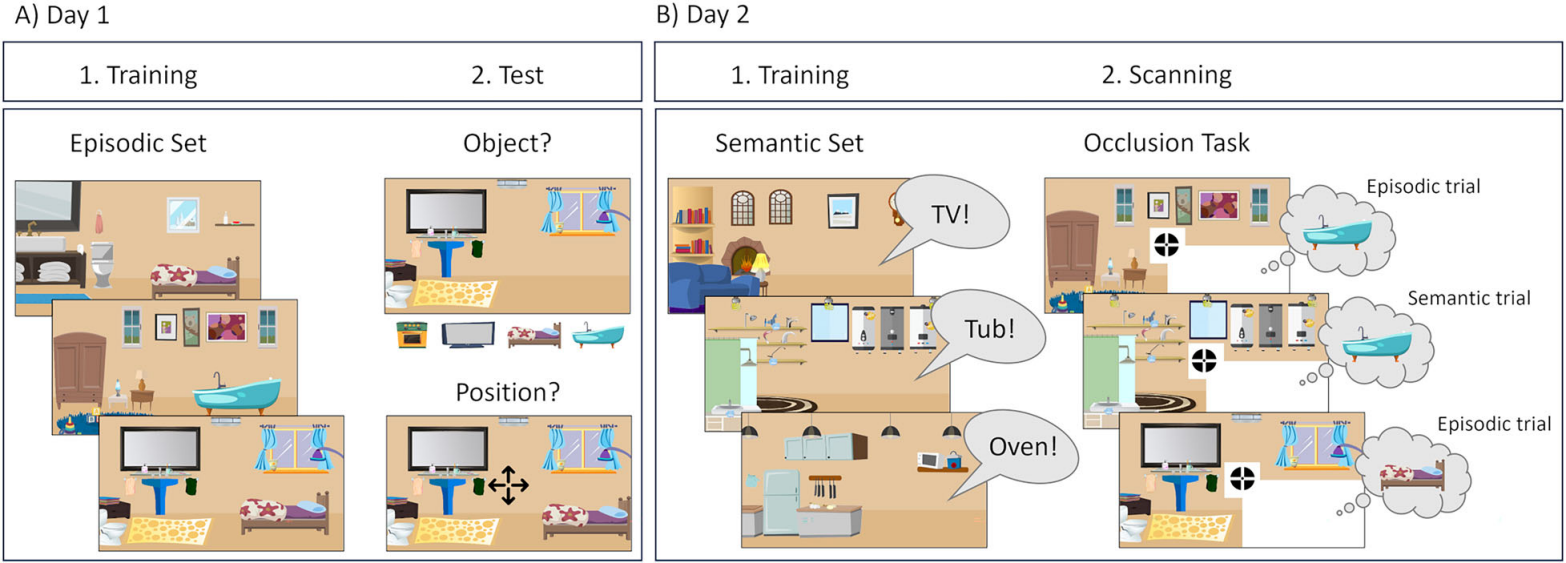
Schematic overview of training and test phases across days. ***A***, During Day 1 training, participants studied room–object combinations with minimal semantic relation, that is, the episodic set. The target objects were always placed in the lower right corner of the room image. After each training cycle, we tested object memory with a 4AFC format, followed by memory for the objects’ original position. ***B***, On Day 2, participants were introduced to the semantic set consisting of new room images and verbally indicated the object with maximal semantic relation to the room. During scanning, the occlusion task showed rooms from both episodic and semantic sets with a white occluder hiding the area of the target object. Participants were asked to focus on the cross in the center of the screen and to reinstate and hold the entire room in mind, including the associated object, with as much detail as possible.

In the learning phase, the participant's task was to study and remember the episodic object–room pairs, that is, combinations with minimal semantic relation (Fig. 1*A*). Younger and older participants underwent five and nine learning cycles, respectively, in which the object–room pairs were presented for 10 s sequentially and repeated ten times. The number of additional learning cycles was piloted to make sure that old adults could reach the threshold of at least 80% accuracy in the final block in order to compensate for the known decline of episodic memory in older adults (Shing et al., 2008). We instructed participants to memorize the object–room combinations and as many details as possible, including the object's exact position in the lower right corner. At the end of each learning block, their memory of the object–room pairings and the object's position was tested. In a 4AFC format, we presented a previously studied room with a white occluder and the four available objects; participants selected one object by pressing a number key, ranging from 1 to 4, on the keyboard with their left hand. We tested the remembered position by presenting the same room with the correct but slightly displaced object and asked the participants to move the object to its original place by pressing the arrow key corresponding to the moving direction with their right hand. While younger adults pressed the keys independently within a 2 s time window, older adults indicated their choice verbally, and the experimenter pressed the keys on their behalf within a 4 s time window. We adjusted the procedure after observing in pilots that some older adults struggled to coordinate choice and response in time.

After completing all learning cycles, we familiarized participants with the structure and timing of the scanner task on Day 2. In this task, the episodic rooms with the occluder were presented sequentially for 4 s each; participants were instructed to fixate on the cross in the center of the screen and to reinstate and hold the room in mind, including the learned object in its original position. After each trial, participants rated the vividness of the retrieved object on a four-point Likert scale. Younger adults entered their ratings via the keyboard, and older adults responded verbally. Finally, all participants conducted another learning block to refresh their memory for the object–room pairs. Unlike in the preregistration announced, participants did not additionally draw the objects on a printed version of the occluded rooms due to time limitations.

### Day 2

The second session took place at the Brain Imaging Center.

#### Prescan phase

Before entering the scanner, we introduced participants to the semantic object–room pairings (Fig. 1*B*), with all rooms being novel to the participants. The eight new room images appeared one after another, always with the occluder, and participants had to name one among the four studied objects with the best semantic fit. The experimenter gave feedback on incorrect object choices. Unlike the episodic object–room pairings, the semantic room images were never directly shown together with the objects to participants. Older adults were exposed to the semantic rooms for two more rounds and received additional practice. In this practice, we presented both episodic and semantic rooms with occluder in random order, and participants had to indicate the correct associated object, which was, depending on the room, semantically or episodically retrieved. We added this practice to ensure that older adults would understand the alternating order of episodic and semantic trials in the occlusion task during scanning and would retrieve the required object depending on the presented room.

#### Scanning phase

The reported structure of the scanning session was identical to the one in Ortiz-Tudela et al. (2023). The scanning sequences were distributed across two blocks of approximately 50 min, with a 10 min break in between these two blocks. The break allowed participants to go to the bathroom and refresh themselves to prevent discomfort and unwanted movements. In Block 1, participants performed two occlusion task runs, a structural scan, and a functional retinotopy run. In Block 2, participants performed two more occlusion task runs, another structural scan, another functional retinotopy scan, and a sensory template run. One additional functional scan and one additional anatomical scan were collected but not used for this project, and thus, they are not discussed further.

##### Occlusion task

Each of the four occlusion task runs presented all episodic and semantic rooms intermixed. We optimized the presentation order using the MATLAB toolbox easy-optimize-x by Spunt (2016) to obtain the most efficient design for detecting activation differences between episodic and semantic trials. A white patch occluded the lower right corner of the rooms, and a fixation cross designed to minimize unwanted eye movements (Thaler et al., 2013) on a small white square covered the foveal region. In each run, all 16 rooms were repeated six times with a presentation duration of 4 s and an intertrial interval of 2 s. Each run lasted 576 s. While the room images flashed at a 5 Hz frequency, the white patches and the fixation cross remained stable, helping the participants focus their gaze on the center of the room. We asked participants not to move, to focus on the fixation cross, and to retrieve the associated object as vividly as possible. When an episodic room (studied on Day 1) was presented, they had to retrieve the object that was studied together with this room on Day 1 (i.e., episodic trial). When a semantic room (introduced on Day 2) was shown, the object with the best semantic fit had to be retrieved (i.e., semantic trial). This procedure ensured the comparability of episodic and semantic trials, with the main difference being the mnemonic retrieval route accessed by the participants.

##### Sensory template

In order to compare feedback with feedforward signals, we ran an additional task in which we showed the 16 correct object–room pairings 12 times (without occlusions) for 1.5 s with an intertrial interval of 1 s. The run lasted 480 s. We optimized the presentation order, as in the occlusion task runs, using easy-optimize-x (Spunt, 2016). Participants fixated on the cross in the center of the screen and performed a one-back task, which served as a cover task to ensure attention was paid to the stimuli. Whenever they detected image repetitions, they had to press a button with the index finger on an MRI-compatible button box.

##### Functional retinotopy and target area mapping

We used standard stimulation procedures to demarcate the early visual cortex (EVC) primary and secondary subfields V1 and V2. For eccentricity mapping, we showed flashing and expanding contrast-reversing checkerboard rings (nine cycles, 56 s/expansion). For polar angle mapping, we showed a flashing and rotating contrast-reversing checkerboard wedge (eight clockwise rotations, 64 s/rotation). Through target area mapping, we identified voxels that topographically represented the lower right corner within areas V1 and V2. To this end, we used two different checkerboard patterns. The first pattern spanned 1° of visual angle along the inner boundary of the occluded lower right corner, and the other pattern covered the remaining inside of the occluded region. Voxels that represented the boundary of the occluder were eventually excluded from further analyses to prevent spillover from adjacent receptive fields and to have a buffer for small misalignments across functional runs (Smith and Muckli, 2010). For further details, see the identical procedure in Ortiz-Tudela et al. (2023).

#### Postscan phase

After both scanning blocks, we asked participants to do one last retrieval task on a laptop outside the scanner. Identical to the memory test for object–room pairings and object position on Day 1, each occluded room was presented together with the four available object options. The presentation order was sequential, with all rooms from the episodic set showing first, followed by all semantic rooms. As on Day 1, younger adults pressed the number and arrow keys themselves with their left hand and within 2 s, and older adults communicated their decision verbally to the experimenter, who pressed the keys on their behalf within 4 s.

### MRI setup and data acquisition

We scanned participants with a 3.0 Tesla Siemens MAGNETOM Prisma scanner with a 32-channel head coil system. 3D structural scans (MPRAGE sequence; resolution, 1 × 1 × 1 mm; iPAT factor = 2) were acquired in both blocks for anatomical reference. EPI sequences (TE = 38 ms; TR = 800 ms; resolution, 2 × 2 × 2 mm; MB factor = 8; flip angle = 52°; field of view = 208 mm; 72 axial slices; phase-encoding direction, AP) were applied to measure the brains’ blood oxygenation level-dependent response. After the first occlusion task run in each block, we acquired five extra volumes for each phase-encoding direction to allow susceptibility distortion correction in EPI sequences.

### Behavioral data analysis

We conducted all behavioral analyses in R (version 4.0.3; R Core Team, 2020) and used the results to explore the data and to identify participants performing below a threshold of 80%.

### fMRI data analysis

#### Preprocessing

Except for the retinotopic and target mapping runs, we preprocessed the fMRI data using functional magnetic resonance imaging data preprocessing pipeline (fMRIPREP) 20.1.1 (Esteban et al., 2019, 2020). fMRIPREP's output provides a CC0-licensed citation boilerplate that includes all preprocessing details. As requested by fMRIPREP's authors, an unchanged copy is available in the corresponding OSF repository. The preprocessing of retinotopic and target mapping runs was conducted in BrainVoyager 21.4 (Brain Innovation) for Linux and included slice time correction, 3D motion correction, and temporal high-pass filtering at 0.01 Hz with linear detrending.

#### ROI definitions

We defined regions of interest as the subset of voxels in the left EVC for V1 and V2, topographically representing the room images’ lower right corner. In this corner, the object was either presented as feedforward visual input (in the sensory template run) or covered (in the occlusion task runs). Covering the corner removed any meaningful feedforward visual stimulation because all the trials in the occlusion task included the same-sized white patch in the identical position. We conducted all further analyses on this particular subset of voxels. We created masks for early visual areas V1 and V2 using standard retinotopic mapping procedures (see Functional retinotopy and target area mapping section) and manual delineation of the subfields. The resulting masks were restricted exclusively to the voxels representing the room's lower right corner using the target mapping run [see Ortiz-Tudela et al. (2023) for a detailed procedure description].

#### Generalized linear model

We extracted single-trial β estimates by applying a least squares separate approach, where each trial is modeled as a separate regressor (Mumford et al., 2012; Abdulrahman and Henson, 2016). For each of the four occlusion task runs, we computed 96 generalized linear models (GLMs). A single GLM comprised 1 regressor for the onset of the current trial, 16 regressors for the onsets of each room, 6 raw head motion regressors (three for displacement and three for rotation), and 3 regressors for global, white matter, and cerebrospinal fluid intensities. For the sensory template run, we conducted 192 GLMs with the same combination of regressor coefficients. In our preregistration, we planned to include six additional nuisance regressors for volume-to-volume eye motion measures in each spatial axis for each eye. We extracted the eye bulbs of each participant using eye state fMRI (Brodoehl et al., 2016) to compute directional vectors for each eye along the anteroposterior axis in a three-dimensional space (*x*, *y*, *z*). However, in our older adult sample, due to large head sizes, the EPI's field of view (208 mm) did not always include a sufficient portion of the eye bulbs to calculate those directional vectors reliably. Thus, to provide comparability of findings across age groups, we decided not to include regressors for eye motion in either sample.

#### MVPA

**Figure 2.**
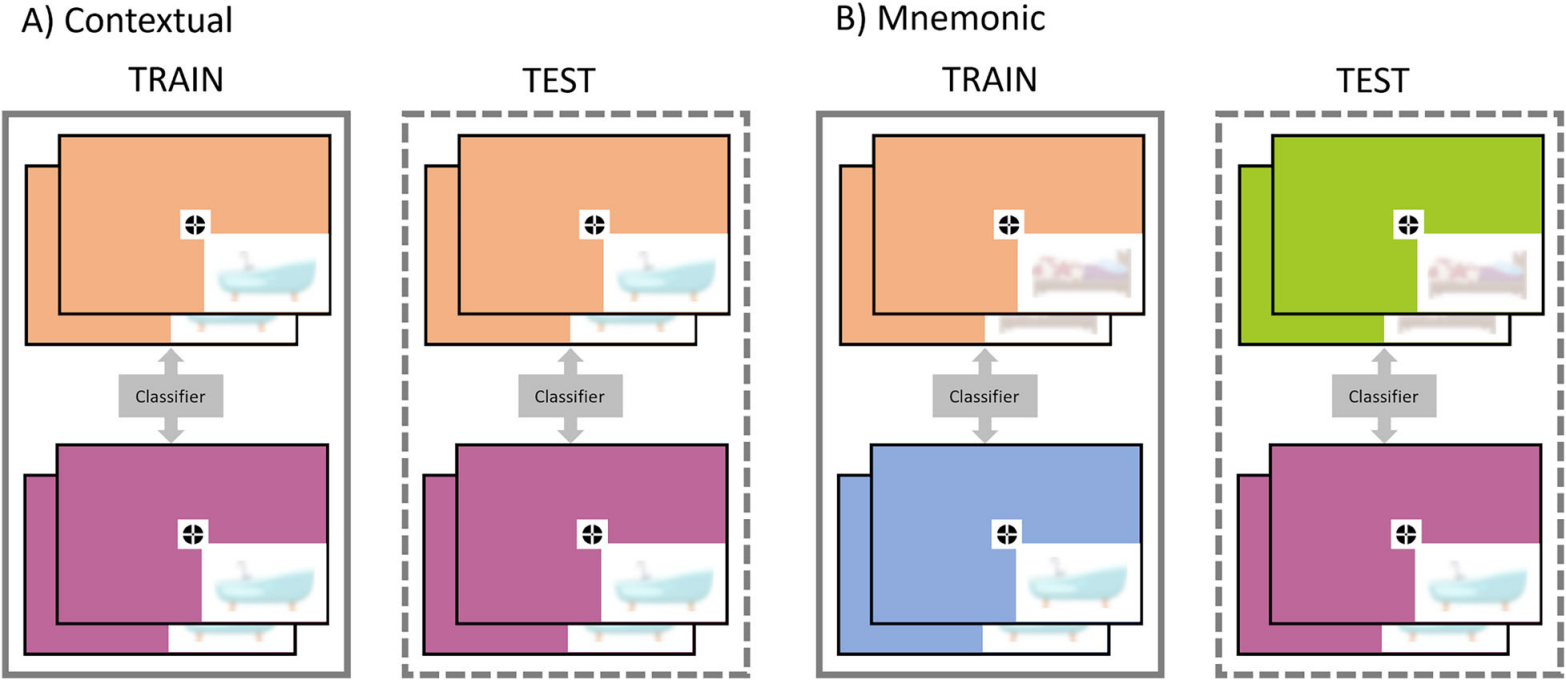
Classification setup. All trials used for decoding analyses stem from the occlusion task performed in the scanner on Day 2. Note that the objects were never shown during the actual task (shown here only for illustration purposes). We performed decoding on voxels in V1 and V2 that represented the occluded lower right corner. ***A***, To decode contextual information, we provided the classifier with a training set of different rooms associated with the same objects (solid box). Based on this arrangement, the classifier could use only the contextual room information in feedback signals to distinguish trials of the upper stack from trials of the lower stack. The test set (dashed box) comprised the same object–room combinations from a separate run from the training set. ***B***, To decode mnemonic information, the classifier was trained with two trial sets of different rooms associated with different objects (solid box). Here the classifier could use contextual and mnemonic feedback to learn to distinguish the upper from the lower stack. When tested on a set of new rooms that shared the same object (dashed box), only the mnemonic feedback that was consistent (across training and test sets) could provide sufficient information to discriminate between them.

We applied MVPA to decode the different components of feedback signals in nonstimulated voxels of V1 and V2 during the occlusion task. We used binary linear support vector machine classifiers with a fourfold leave-one-run-out cross-validation procedure. We trained classifiers on three of four occlusion task runs (288 trials) and tested on the remaining run (96 trials). We repeated this train–test procedure for all four runs and averaged the resulting classification accuracies across folds. All classification analyses were performed separately for episodic and semantic trials. Figure 2 illustrates the classification schemes we adopted to decode the specific feedback components, that is, contextual and mnemonic. Contextual refers to the visual information provided by the room image surrounding the occluded region. This contextual information is fed through lateral connections to the adjacent nonstimulated receptive fields, where it can be used to disambiguate the percept and aid the retrieval of the associated object. To capture contextual information in V1 and V2, we trained a classifier with and tested on “same object–different room” combinations (i.e., the two class labels share the object but differ in the room) so that only contextual feedback could provide the classifier with information to discriminate between room and object. Mnemonic refers to the object information retrieved through an episodic or semantic route and transmitted to nonstimulated receptive fields in V1 and V2. We trained another classifier based on a cross-classification schema to decode mnemonic information. The training set consisted of “different object–different room” combinations (i.e., the two class labels neither share the object nor the room), in which the classifier learned to discriminate between object and room using both feedback information types. A classifier tested in this set could use either (or both) the object or the room to discriminate the classes. However, when tested on a different subset of rooms that shared the same objects across training and test sets, above-chance classification could be achieved only by relying on the mnemonic object information. We chose this more conservative cross-classification schema as it prevents using any learned room information and enables classification solely on object information. Note that an alternative classification schema would be training a classifier with and testing on “different object–same set of rooms” combinations. However, this combination was not part of the experimental design and would lead to interference if a particular room cues two different objects. We performed all decoding analyses with The Decoding Toolbox (Hebart et al., 2015). We averaged classification estimates across participants and tested for significance using a two-step bootstrapping approach (Stelzer et al., 2013). An accuracy distribution was created for each participant by randomly permuting the trial labels 100 times and calculating classification accuracies for each iteration. We drew a random sample (with replacement) from each distribution and averaged across participants 1,000 times, thus creating a null distribution of 1,000 average accuracies. If classification estimates were larger than 99.9% of the accuracies in the null distribution (*p* < 0.001), they were considered significant. For age group comparisons, we used linear mixed models (LMMs, lmer function in the *lme4* package; Bates et al., 2015) instead of ANOVAs, as written in the preregistration, to control for additional variance attributed to participants. Age group (older vs younger), trial type (episodic vs semantic), and ROI (V1 vs V2) were included as predictors, and random intercepts were specified per subject. For main effect testing, we calculated type II Wald *F* tests using the ANOVA function in the *car* package (Fox and Weisberg, 2019) and type III Wald *F* tests for interaction testing. Confidence intervals were determined using the confint function from the *stats* package (R Core Team, 2020).

#### Representational similarity analysis

For each participant, we used single-trial β estimates to compute representational dissimilarity matrices (RDMs) between every pair of individual trials (Kriegeskorte et al., 2008). We calculated all RDMs using The Decoding Toolbox (Hebart et al., 2015). As distance measures, we used the cross-validated Mahalanobis distance for the occlusion task RDMs and Pearson’s *r* for the single-run sensory template RDMs.

#### Model correlations

**Figure 3.**
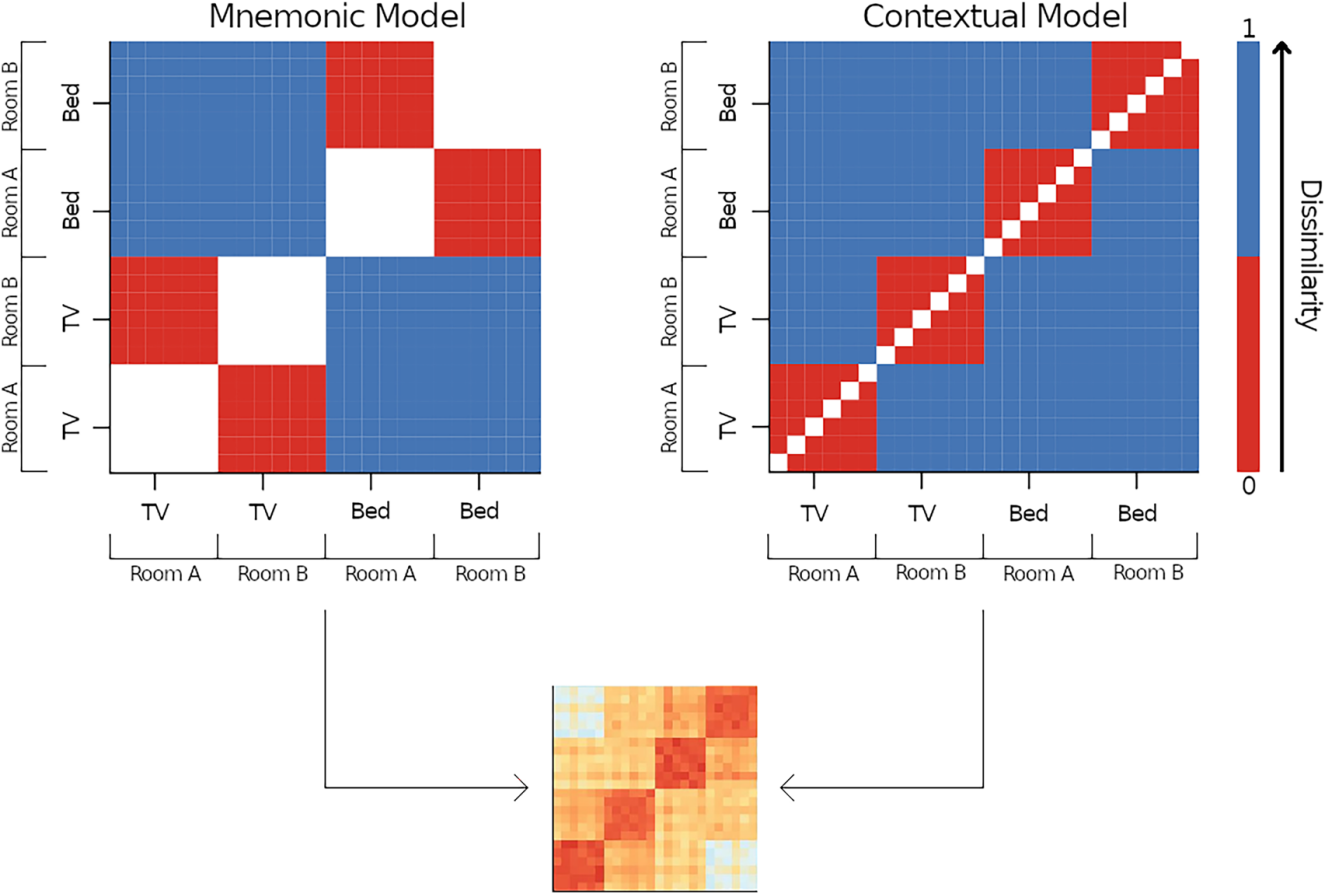
Model RDMs. Two model RDMs representing an ideal neural response pattern were created and correlated with the neural RDM of a participant. For illustration purposes, reduced model matrices spanning 24 × 24 trials instead of 96 × 96 trials are plotted. Cells represent four object–room pairings (TV-Room A, TV-Room B, Bed-Room A, Bed-Room B) containing either zeros (lowest possible dissimilarity) or ones (highest possible dissimilarity). In the left panel mnemonic model, “same object–different room” cells are colored in red, indicating zeros since retrieving the same object should ideally result in the lowest possible dissimilar neural signal. “Different object–different room” cells are colored in blue, indicating ones since retrieving different objects should ideally result in the highest possible dissimilar neural signal. “Same object–same room” cells are colored in white and were removed from the correlation analysis to prevent the coefficient from being artificially lowered through room similarity. In the right panel contextual model, “same object–same room” cells are colored in red, indicating zeros since perceiving the same room images should ideally result in the lowest possible dissimilar neural signal. “Same object–different room” cells are colored in blue, indicating ones since perceiving different rooms should ideally result in the highest possible dissimilar neural signal. The diagonal cells are colored in white and were removed from the correlation analysis to prevent the coefficient from being artificially lowered through overall similarity.

To investigate to what extent contextual and mnemonic information is represented in feedback signals within both ROIs, we created two model RDMs that reflected the ideal correlation pattern for each feedback component (Fig. 3). Both model matrices were equally sized as the individual neural RDMs, spanning 96 × 96 trials (48 episodic and semantic trials, respectively). The contextual model had zero values for “same object–same room” cells because we expected those combinations to have the lowest dissimilarity and maximal values (i.e., ones) for the remaining high dissimilarity cells. The diagonal, containing only zeros, was excluded from all analyses. In the mnemonic model, we expected the lowest dissimilarity for “same object–different room” cells, therefore having zero values. “Different object–different room” cells had maximal values representing the highest dissimilarity. “Same object–same room” cells were removed as the low dissimilarity between the same rooms would artificially reduce the final dissimilarity measure, which should be solely based on the object. Both models were Spearman rank correlated with all individual RDMs. Correlating the dissimilarity matrices results in correlation values that can range from zero to one, with low values representing low similarity between the model and individual RDMs and high values representing high similarity between the model and individual RDMs. The correlation coefficients were Fisher’s *z* transformed and compared against zero using Wilcoxon signed-rank tests (wilcox.test function from *stats* package, R Core Team, 2020). As for MVPA, we performed age comparisons using LMMs with random intercepts per participant and model (object vs room), trials (episodic vs semantic), and age (older vs younger) as predictors.

#### Differentiation index

Similar to the procedure in Koen et al. (2019), we calculated differentiation indices (DIs) to obtain a measure of the specificity of neural responses. For the DI calculation, we used the dissimilarity values from the individual neural RDMs and subtracted the average within from the average between dissimilarities. Within dissimilarity refers to the pairwise distance of trials that share the same object, for example, TV versus TV, whereas between dissimilarity refers to the pairwise distance of trails comprising different objects, for example, TV versus bathtub. Thus, well-preserved neural differentiation is represented by higher DIs resulting from lower within and higher between dissimilarities. We computed DIs for each participant, occlusion task trials (episodic or semantic), and ROI. We followed the same procedure for the sensory template runs. DIs for the occlusion task are henceforth referred to as feedback DIs, and DIs for the sensory template run are referred to as feedforward DIs. We contrasted the resulting indices against zero with one-sided Wilcoxon tests and conducted age group comparisons separately for feedforward and feedback DIs using LMMs with age (older vs younger), trial type (episodic vs semantic), and ROI (V1 vs V2) as predictors and random intercepts per subject.

#### Correlation of feedforward and feedback DIs

We transformed all feedforward and feedback DIs into *z* scores. Note that a single feedforward DI was computed per participant and ROI as the one-back task in the sensory template run did not include a distinction between episodic and semantic trials. Outliers were defined as DIs above or below 3.29 standard deviations (signaling the most extreme 0.1%) from the mean and excluded from further analyses. For younger adults, we removed two feedforward and two feedback outliers from the DI data of ROI V2, and for older adults, we excluded two feedforward and one feedback outlier from the DI data of ROI V1. Finally, we Spearman correlated (one-sided) the average feedforward DIs with episodic and semantic feedback DIs separately for age groups and ROIs. All *p*-values were adjusted using the Benjamini and Hochberg correction (Park et al., 2010).

## Results

### Behavioral results

#### Training performance on Day 1

**Figure 4.**
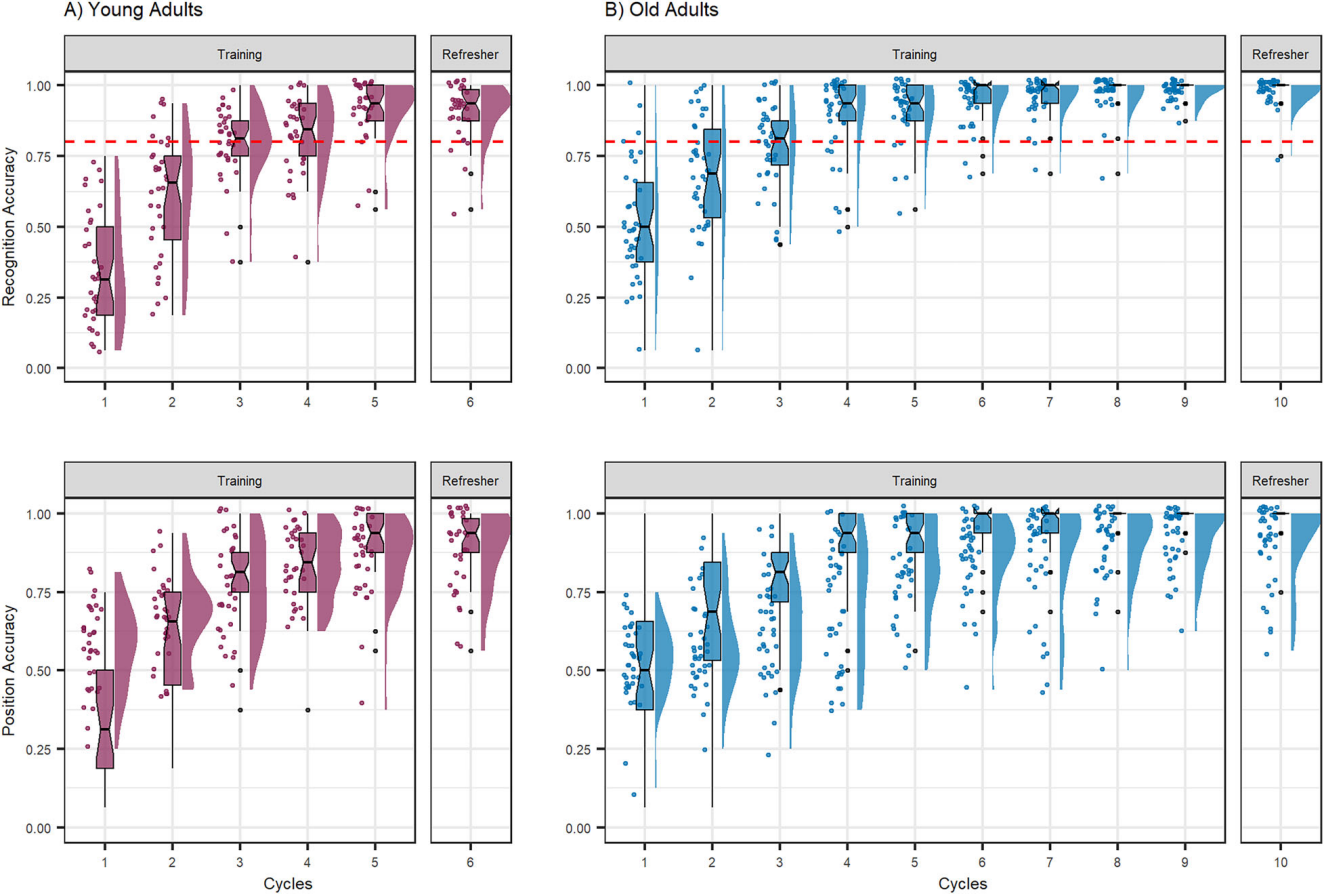
Memory performance across learning cycles on Day 1. The proportion of correct responses for object recognition (upper panel) and position memory (lower panel) across five learning cycles for younger (left panel) and nine learning cycles for older adults (right panel), plus one refresher cycle at the end of the session, respectively. The dashed red line indicates the threshold of 80% that had to be crossed, either in the last or the refresher cycle of the object recognition task, to be included for the fMRI analysis.

Both age groups learned the object–room associations successfully across their designated number of learning cycles, that is, five for younger and nine for older adults, plus one refresher cycle at the end of the session. All participants crossed the threshold of at least 80% learning performance either in the last training or in the refresher cycle at the latest. Figure 4 shows the learning progress of both age groups for object recognition and object position memory, respectively.

#### Postscan memory performance on Day 2

After being scanned, we tested participants’ memory for object–room associations and object position to ensure they maintained the required retrieval performance during scanning. On average, both age groups performed above 80% in both trial types (episodic, *M*_younger_ = 0.81, SD_younger_ = 0.16; *M*_older_ = 0.86, SD_older_ = 0.16; semantic, *M*_younger_ = 0.84, SD_younger_ = 0.15, *M*_older_ = 0.95, SD_older_ = 0.07). Unpaired two-sided *t* tests showed no differences across age groups in episodic trials for either object memory (*t*_(68.49)_ = −1.43, *p* = 0.156) or object position (*M*_younger_ = 0.77, SD_younger_ = 0.18, *M*_older_ = 0.78, SD_older_ = 0.21, *t*_(69.95)_ = −0.24, *p* = 0.805). The mean recognition memory performance for objects from semantic trials was better for older compared with younger participants (*t*_(43.77)_ = −3.61, *p* < 0.001).

### fMRI results

The previous study with only younger adults (Ortiz-Tudela et al., 2023) showed that contextual and mnemonic feedback signals contributed to the activation pattern in nonstimulated voxels of the primary and secondary visual cortices V1 and V2. Interestingly, the extent to which mnemonic feedback signals fed down to these early cortices depended critically on the retrieval route. That is, only episodic mnemonic content was represented but not semantic mnemonic content. This difference in content was revealed through representational similarity analysis (RSA), which was conducted after observing that the initial MVPA decoding approach was only sensitive to contextual information and failed to capture mnemonic information.

Notably, the lifespan trajectories of episodic and semantic memory are different; semantic memory remains relatively stable across age with later and less decline than episodic memory (Li et al., 2004; Rönnlund et al., 2005). We, therefore, hypothesized that the individual components of feedback would also change with age. More concretely, we expected that the amount of episodic feedback would be reduced in older adults. Semantic feedback, on the contrary, should be less affected by age-related changes as semantic memory content tends to be relatively preserved in older age (Nyberg et al., 2003, 2012; Haitas et al., 2021). Hence, we anticipated less or no decay in semantic feedback signals compared with episodic feedback signals. For contextual feedback signals, we expected no substantial differences between age groups because the contextual visual input was identical and immediately available, and any visual impairment was corrected for all participants with appropriate MRI-compatible glasses.

#### Decoding contextual (and mnemonic) feedback signals

Following the previous study by Ortiz-Tudela et al. (2023), we addressed our hypothesis first with a classifier-based approach. We set up two classification schemes to decode contextual and mnemonic information, respectively, from nonstimulated voxels of V1 and V2 (for details about classifier arrangements, see Materials and Methods, MVPA). The first classifier arrangement aimed at capturing contextual room information. In both age groups, the classifier performed above the chance level (0.50) for both episodic and semantic trials in both ROIs (younger adults, V1_epi_ = 0.69, V2_epi_ = 0.68, V1_sem_ = 0.65, V2_sem_ = 0.66; older adults, V1_epi_ = 0.58, V2_epi_ = 0.58, V1_sem_ = 0.58, V2_sem_ = 0.58, all *p*'s < 0.001; one-sided one-sample *t* test). LMM analysis revealed a significant main effect of age on contextual room information, with classification accuracy in older adults being lower than in younger adults [*β* = −0.077%, 95% CI (−0.111, −0.043), *t* = −4.424, *p* < 0.001]. These results replicate and extend previous findings by showing that contextual information is a reliably traceable constituent of feedback signals in nonstimulated voxels of early visual areas not only in younger but also in older adults. Although we did not anticipate a significant age effect on contextual feedback, the result is in line with some studies that found an age-related decrease in neural specificity within feedback signals (St-Laurent et al., 2014; Trelle et al., 2019).

The second classifier arrangement aimed at capturing mnemonic object information. The classifier did not perform above the chance level (0.50) in younger or older adults. Therefore, we were unable to look further into age comparisons (younger adults, V1_epi_ = 0.49, V2_epi_ = 0.47, V1_sem_ = 0.49, V2_sem_ = 0.49; older adults, V1_epi_ = 0.51, V2_epi_ = 0.51, V1_sem_ = 0.50, V2_sem_ = 0.51, all *p*'s > 0.05, one-sided one-sample *t* test). This classification failure in older adults replicates the previous study's finding with younger adults (Ortiz-Tudela et al., 2023). Nevertheless, this null result does not rule out the possibility of mnemonic object information existence in feedback signals. We reasoned that the classifier might have failed to decode object information at the test because it might have learned to classify primarily based on room information and consequently could not generalize its’ knowledge to a test set of new rooms.

#### Coexistence of contextual and mnemonic feedback signals revealed by RSA

**Figure 5.**
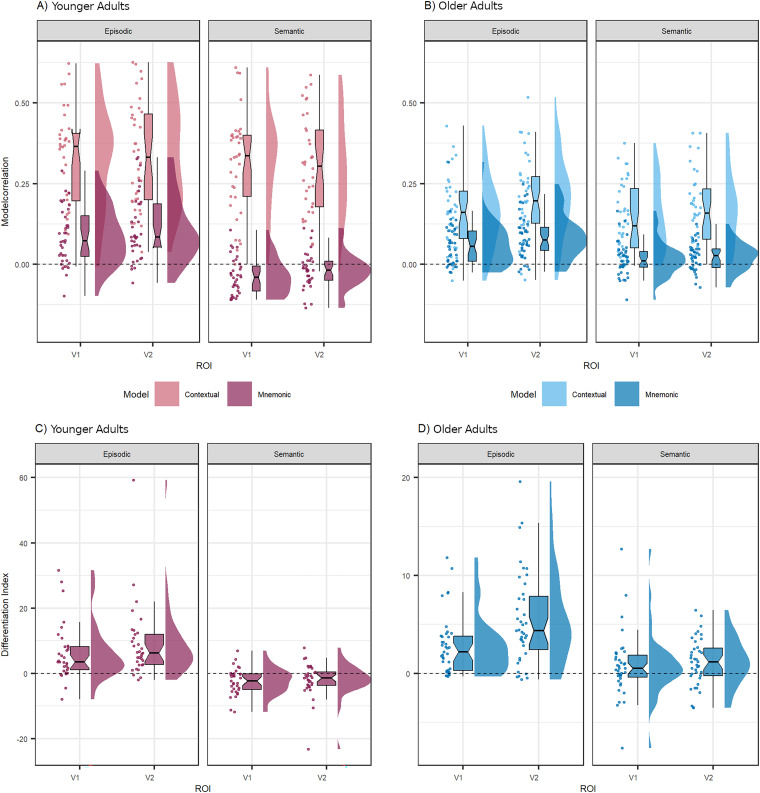
Model correlations and feedback DIs. The top panel shows the Spearman correlations (*z* scores) for (***A***) younger and (***B***) older participants between individual RDMs and two model RDMs representing ideal dissimilarity correlation patterns for context (contextual model) and object categories (mnemonic model), respectively. Correlations are separately shown for episodic and semantic trials in ROIs V1 and V2. The bottom panel shows the DIs separately for episodic and semantic mnemonic feedback for (***C***) younger and (***D***) older participants in both ROIs V1 and V2. The dashed vertical line indicates zero.

Similar to the previous study, we addressed the null result for decoding mnemonic information by using RSA, which enabled the identification of different sources of variance within the same data (Ortiz-Tudela et al., 2023). We correlated individual RDMs from episodic and semantic trials of both ROIs with two model RDMs that represent ideal dissimilarity correlation patterns for the room (contextual model) and object categories [mnemonic model; see Materials and Methods, Representational similarity analysis for further details about robust decision-making (RDM) model specifications]. Figure 5, *A* and *B*, shows the correlations with both model RDMs for younger and older adults, respectively. Correlating the contextual room model with episodic RDMs resulted in a moderate relationship for younger (rho_V1_ = 0.31, rho_V2_ = 0.33, both *p*'s < 0.001) and a weaker relationship for older participants (rho_V1_ = 0.15, rho_V2_ = 0.20, both *p*'s < 0.001). We observed a similar age pattern in the correlations between the contextual room model with semantic RDMs: moderate for younger and weaker for older participants (younger, rho_V1_ = 0.31, rho_V2_ = 0.30, both *p*'s < 0.001; older, rho_V1_ = 0.14, rho_V2_ = 0.16, both *p*'s < 0.001). Interestingly, correlating the mnemonic model with episodic RDMs resulted in a low positive relationship for both age groups (younger, rho_V1_ = 0.08, rho_V2_ = 0.11, both *p*'s < 0.001; older, rho_V1_ = 0.06, rho_V2_ = 0.08, both *p*'s < 0.001), whereas correlating the mnemonic model with semantic RDMs resulted in different relationships for the two age groups. In younger adults, a low negative relationship emerged (rho_V1_ = −0.03, *p* < 0.001, rho_V2_ = −0.01, *p* = 0.009), but in older adults, the correlation turned out positive (rho_V1_ = 0.01, *p* = 0.037, rho_V2_ = 0.02, *p* < 0.001).

Testing these observations formally, LMM analysis revealed a significant three-way interaction between model, trial type, and age [*β* = −0.093%, 95% CI (−0.183, −0.003), *t* = −2.023, *p* = 0.043]. We further investigated the interaction by running LMMs separately for the two models. For the contextual model, only a main effect of age emerged [*β* = 0.153%, 95% CI (0.114, 0.191), *t* = 7.793, *p* < 0.001] but no significant interaction between age and trial type [*β* = 0.006%, 95% CI (−0.046, 0.059), *t* = 0.252, *p* = 0.800]. In particular, older adults, compared with younger adults, showed lower correlations between the contextual model with both episodic and semantic RDMs. For the mnemonic model, we found a significant interaction between age and trial type [*β* = −0.073%, 95% CI (−0.103, −0.044), *t* = −4.859, *p* < 0.001], indicating that the difference between younger and older age groups was larger in semantic trials (*t*_(276)_ = 4.42, *p* < 0.001) than in episodic trials (*t*_(276)_ = −2.37, *p* = 0.018), specifically because younger adults showed lower correlation estimates in the negative direction, while older adults showed higher, positive correlation estimates.

Taken together, these findings are in line with our classification results by showing that (1) contextual information is present in feedback signals in both age groups, trial types, and ROIs and (2) the amount of contextual information is overall reduced in older adults early visual areas V1 and V2, suggesting dedifferentiation of contextual feedback signals. In contrast to our classification results and in line with the original study with younger adults only, RSA revealed that mnemonic information exists in both younger and older adults’ primary and secondary cortices. Interestingly, episodic feedback was comparably well reinstated in younger and older adults, whereas semantic feedback was more reliably reinstated in older but not in younger participants. Even though these results are contrary to our expectations, they lend support to previous research showing that older adults rely on semantic knowledge more extensively, especially benefiting from it when learning new information in line with their prior knowledge (Badham and Maylor, 2014; Mohanty et al., 2016; Lalla et al., 2022).

In our preregistration, we included a variance partitioning approach to further explore the unique contribution of different sources of information on activation patterns, particularly the age differences therein. However, in observation of dedifferentiation, the reduced contextual feedback results indicated a higher noise level in older adults’ brains. Thus, the amount of variance that could be explained is presumably lower in older adults and, consequently, not comparable to the amount of variance available in younger adults. Consequently, we did not pursue this analysis to prevent inappropriate interpretations when comparing the amount of variance between age groups.

#### Dedifferentiation in mnemonic feedback signals and feedforward visual input

So far, we have provided empirical evidence for the existence of contextual and mnemonic feedback signals not only in younger but also in older adults. We further showed that the composition of feedback signals changes over the lifespan. Compared to younger adults, contextual feedback was reduced, episodic feedback was similar, and semantic feedback was stronger in older adults. To further characterize dedifferentiation in mnemonic feedback (i.e., specificity in object information), we calculated DIs for each trial type and ROI. DIs were tested against zero and compared across age groups. Following previous research on age-related episodic memory decline, we expected primarily episodic feedback to suffer from age-related dedifferentiation; that is, older adults would have lower DIs in episodic trials than younger adults.

Figure 5, *C* and *D*, shows feedback DIs for younger and older adults, respectively. Feedback DIs for both age groups were significantly different from zero (younger adults, V1_epi_ = 25.34, *z* = 4.43, *p* < 0.001; V2_epi_ = 22.05, *z* = 5.64, *p* < 0.001; V1_sem_ = −3.87, *z* = −3.30, *p* < 0.001; V2_sem_ = −3.69, *z* = −2.29, *p* = 0.010; older adults, V1_epi_ = 10.71, *z* = 5.70, *p* < 0.001; V2_epi_ = 3.41, *z* = 6.09, *p* < 0.001; V1_sem_ = 0.50, *z* = 1.65, *p* = 0.049; V2_sem_ = 1.15, *z* = 2.74, *p* = 0.002; one-sided Wilcoxon test). LMM analyses resulted in a significant two-way interaction between age and trial type [*β* = −6.75466%, 95% CI (−10.521, −2.969), *t* = −3.478, *p* < 0.001]. The difference between age groups was higher in semantic trials (*t*_(284)_ = 3.21, *p* = 0.001) compared with episodic trials (*t*_(284)_ = −3.61, *p* < 0.001), with younger adults having lower semantic DIs than older adults. This finding is in line with the RSA model correlation results suggesting that episodic feedback does not show compromise in neural specificity in older age. Interestingly, semantic feedback signals even increased in DIs, such that the neural specificity for this mnemonic content improves in older age. To complement those findings, we explored neural differentiation within our ROIs when the objects were presented as feedforward visual input during the sensory template run (i.e., episodic and semantic object–room pairings were consecutively presented during scanning; for details, see Materials and Methods, Sensory template). All feedforward DIs were different from zero (younger adults, V1_epi_ = 0.021, *z* = 6.08; V2_epi_ = 0.014, *z* = 5.64; V1_sem_ = 0.024, *z* = 6.23; V2_sem_ = 0.012, *z* = 5.56; older adults, V1_epi_ = 0.009, *z* = 5.25; V2_epi_ = 0.009, *z* = 5.65; V1_sem_ = 0.006, *z* = 4.10; V2_sem_ = 0.005, *z* = 4.42; all *p*'s < 0.001, one-sided Wilcoxon test). Through LMM analysis, we observed a significant main effect of age on neural differentiation [*β* < 0.001%, 95% CI (0.009338798, 0.027471357), *t* = 3.936, *p* < 0.001], meaning that feedforward DIs were lower in older compared with younger adults. Therewith, we replicated previous research and contributed additional evidence for age-related neural dedifferentiation at the item level in the early visual areas V1 and V2.

#### Relating feedforward and feedback components across age

**Figure 6.**
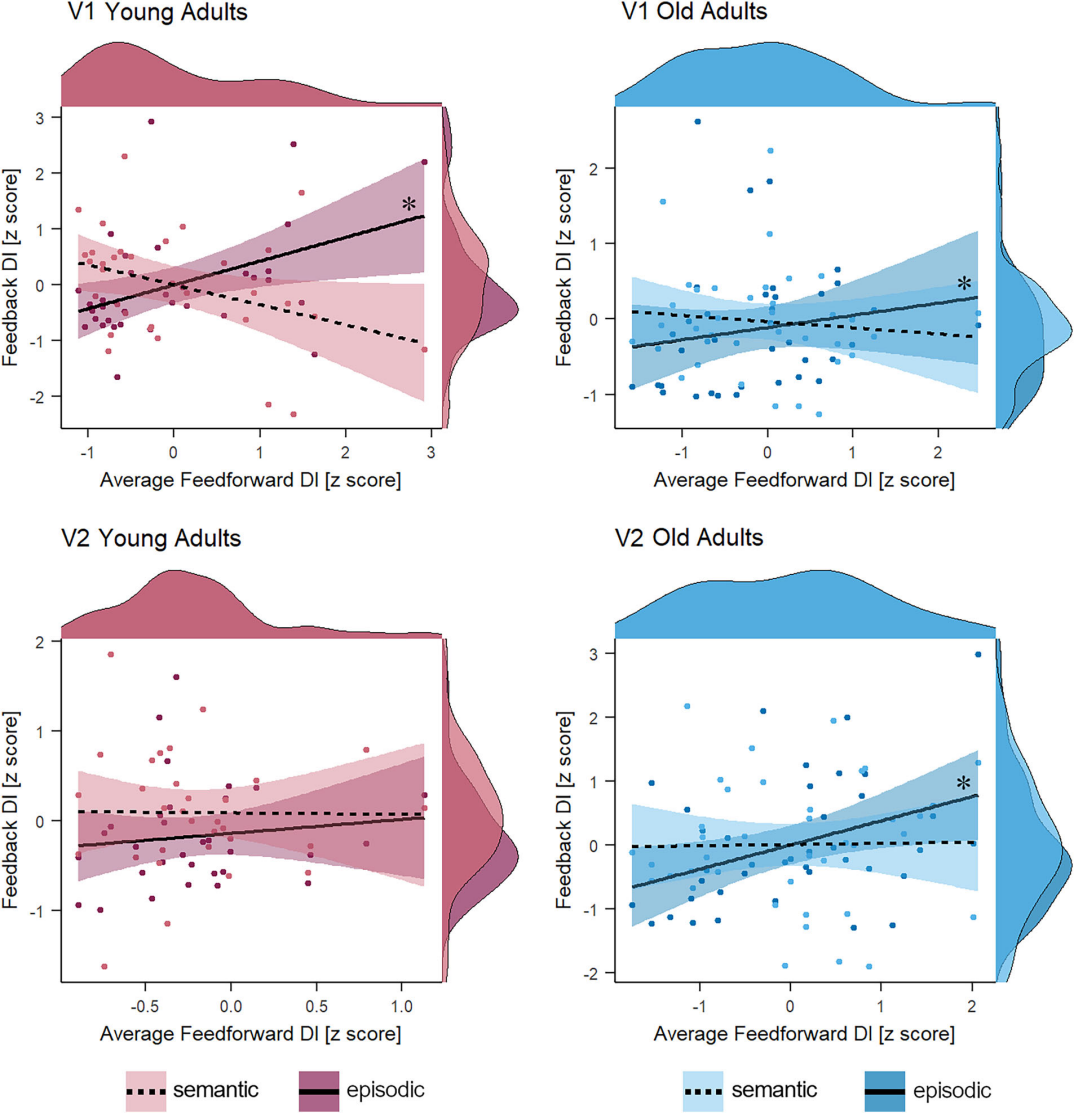
Relationship between visual feedforward and mnemonic feedback DIs. Spearman correlations between average feedforward DIs and episodic and semantic feedback DIs are plotted for younger (left panel) and older adults (right panel) and separately for ROIs V1 (top) and V2 (bottom). The darker colors and solid lines represent the episodic feedback DIs, and the lighter colors and dashed lines represent the semantic feedback DIs. The shaded ribbons represent the 95% CI. Average feedforward DIs were positively related to episodic feedback DIs in younger adults’ V1 and older adults’ V1 and V2.

It has been suggested that dedifferentiation could result from a general age-related-deficient dopaminergic modulation (Li et al., 2001; Abdulrahman et al., 2017). If the underlying mechanism for age-related dedifferentiation is general across brain areas and pathways, feedforward and feedback signals might be impacted to a comparable extent. Based on this assumption, we expected to find a positive relationship between neural specificity in feedforward and individual feedback components as age increases. To this end, we correlated the feedforward DIs with DIs for episodic and semantic feedback separately per age group and ROI. The correlation between feedforward and semantic feedback DIs (Fig. 6) neither resulted in a significant relationship in older (rho_V1_ = −0.11, *p* = 0.736, rho_V2_ = 0.11, *p* = 0.334) nor in younger adults (rho_V1_ = −0.3, *p* = 0.179, rho_V2_ = −0.05, *p* = 0.616). A different picture emerged when we correlated feedforward with episodic feedback DIs. In older adults, we observed a low positive relationship between feedforward and episodic feedback DIs in V1 and V2 (rho_V1_ = 0.34, *p* = 0.044, rho_V2_ = 0.35, *p* = 0.044), whereas, in younger adults, this positive relationship appeared only in V1 (rho_V1_ = 0.41, *p* = 0.035, rho_V2_ = 0.18, *p* = 0.215). This pattern supports the hypothesis that the putative mechanism of dedifferentiation (e.g., dopaminergic modulation) impacts both feedforward and feedback signals but points out that this is only true for a specific component of the feedback signal. Specifically, mnemonic content retrieved through an episodic route was especially prone to age-related changes in the neural mechanism that fosters dedifferentiation, while semantic content was spared. Furthermore, this result implies that certain brain areas, such as V1, are more affected by dedifferentiation than others.

## Discussion

The present study examined age-related changes in the composition of feedback signals in the early visual areas of younger and older adults. We combined an occlusion paradigm with multivariate fMRI pattern analysis, allowing us to isolate and examine concurrent contextual and time-distant mnemonic information in feedback signals in V1 and V2. As the first study that scrutinized age-related differences in feedback signals, four main findings emerged.

First, concurrent contextual and time-distant mnemonic information coexist as feedback signals in V1 and V2 of both younger and older adults. By this, we replicated previous research that identified contextual (Smith and Muckli, 2010; Muckli et al., 2015) and mnemonic (episodic or semantic) information in feedback signals (Ortiz-Tudela et al., 2023) in younger adults’ visual cortex occluded from feedforward visual input and extended this finding to older adults. As occlusions are ubiquitous in everyday life, both young and senescent visual systems must overcome the perceptual challenge of disambiguating uncertain visual input. Feedback signals facilitate this process by carrying information from the concurrent surroundings and internally retrieved time-distant memory representations to “fill in the blank” in the case of occlusion.

Second, the decoding accuracy of contextual feedback was reduced in older adults’ V1 and V2. Lower correlations between a contextual feedback model and multivariate activation patterns in older adults supported this finding. Reduced classification accuracy and (dis)similarity measures have been previously interpreted to indicate age-related dedifferentiation in neural representations (Abdulrahman et al., 2017; Trelle et al., 2019; Folville et al., 2020). Our results showed that contextual feedback is prone to age-related dedifferentiation. Common age-related changes in the neural circuitry within the visual cortex could account for this reduction. For example, demyelination, reduced spine, and synapse densities may lead to dendritic and axonal regressions, which may hamper the integrity of neural signal that is transferred via lateral intracortical connections to adjacent receptive fields (Smith and Muckli, 2010; Larkum, 2013; Danka Mohammed, 2021). However, the exact nature and impact of age-related decline in microstructure integrity within early visual regions is not fully known and needs to be examined in future research. Additionally, an increased baseline noise level in older adults’ EVC has been shown to compromise perceptual processing (Li et al., 2001; Tran et al., 2020). In line with this, we found feedforward DIs to be significantly lower in older adults, probably rendering the transmitted contextual feedback less representative of the original input. Future studies should consider including diffusion tensor imaging to obtain measures for the structural integrity of lateral neural connections to account for such changes (Voss et al., 2008).

Despite the compromised lateral transfer of contextual information, the representational quality of mnemonic feedback could nevertheless be preserved due to compensatory mechanisms (Park et al., 2001). For example, older adults have been shown to recruit more neural resources at low task demand levels as a compensatory strategy, improving neural distinctiveness (Reuter-Lorenz and Cappell, 2008; Carp et al., 2010). This observation may be important to consider with our third main finding: episodic feedback remained well differentiated across age groups, whereas semantic feedback was even better differentiated in older adults than in younger adults. The maintenance of episodic feedback signals was unexpected for several reasons. Age-related episodic memory decline is well established in the aging literature, as well as the notion that older adults tend to retrieve only the gist of a previously experienced episode; namely, contextual details are lost while the central aspects are preserved (Koutstaal and Schacter, 1997; Old and Naveh-Benjamin, 2008; Nyberg et al., 2012; Abadie et al., 2021). Interestingly, while there is evidence for reduced episodic memory reinstatement in the visual cortex (Zheng et al., 2018), some studies found neural reinstatement of episodic memory content as age-invariant (Wang et al., 2016; Thakral et al., 2017, 2019). According to the *Lifetime Experience Hypothesis* by Koen and Rugg (2019), the absence of age-related dedifferentiation could be explained by high familiarity with the stimulus material in both age groups, attenuating differences in neural specificity of episodically retrieved feedback. Furthermore, our training procedure might have contributed to the well-differentiated episodic feedback, which was also mirrored by very high postscan recognition memory performance for episodic object–room combinations. Older adults received four additional learning cycles for encoding the episodic set on Day 1. These additional cycles, together with the relatively small training set (eight object–room combinations), could have provided older adults the opportunity to compensate for any attentional or binding deficits during encoding and eventually diminished effects of dedifferentiation, leading to a comparable behavioral performance (for a similar pattern, see St-Laurent et al., 2014). Furthermore, we assume that due to our recruiting strategy through the university, the older adults were positively biased, characterized by youth-like memory integrity and distinctiveness of neural representations (Fandakova et al., 2015; Zhang et al., 2020; Katsumi et al., 2021). Taken together, our results show that episodic feedback is not compromised when performance level is matched between age groups.

In terms of semantic feedback, we expected no age difference or only slightly less differentiation in older compared with younger adults. Somewhat surprisingly, our results showed that semantic feedback was even better differentiated in older than younger adults. This finding supports studies suggesting that the semantic memory system remains relatively intact in older adults, even with improvements in some domains (Laumann Long and Shaw, 2000; Levine et al., 2002; Lalla et al., 2022). Interestingly, while most studies investigated dedifferentiation using episodic retrieval tasks, the only study that employed an additional semantic task did not find neural dedifferentiation, pointing to the preservation of generalized knowledge in older age (St-Laurent et al., 2011). Moreover, the ecological validity of our stimulus material, as well as the congruity between semantic object–room pairs, might have helped older adults at retrieval and thereby enhanced neural differentiation of semantic feedback (Badham and Maylor, 2014; Mohanty et al., 2016; Brod and Shing, 2019). Although the semantic set was not as extensively trained as the episodic set, the semantic postscan retrieval performance was better in older adults than younger adults, which underlines the growing impact of semantic knowledge over the lifespan and supports previously reported relationship between higher neural differentiation and better memory retrieval (Katsumi et al., 2021). Nevertheless, it should also be noted that the poorer postscan memory performance of younger adults for semantic trials could have been a by-product of the different response formats between age groups. Younger adults entered their responses with their left hand within a 2 s time window, potentially leading to some premature response errors (note that two errors would already result in a performance level of 75%). In contrast, older adults verbally indicated the objects within 4 s, and the responses were entered by the experimenter.

In addition to the growing relevance of semantic memory, a decline in inhibitory control might challenge older adults to suppress accessing semantic information, which happens largely automatically (Karl Healey et al., 2013; Vachon et al., 2019). At the same time, intensified recruitment of semantic knowledge could compensate for deterioration elsewhere, such as feedforward perception and contextual feedback. Future research should employ longitudinal study designs to track changes in feedback signals as they may emerge gradually over the lifespan. To further scrutinize the sources and compensatory mechanisms that may underlie age-related changes in feedback composition, connectivity analysis techniques such as psychophysiological interaction analysis or deep neural networks could be utilized (cf. Deng et al., 2021; Ortiz-Tudela et al., 2023).

Fourth, while differentiation of feedforward visual input was overall reduced in older adults, the extent of differentiation was positively related to episodic feedback signals in both older adults’ V1 and V2 and younger adults’ V1. That is, the retrieval of better-differentiated information through an episodic route correlated with better differentiation of visual input in early visual areas across age groups. This result partly met our hypothesis predicting a positive relationship between neural differentiation in feedforward and feedback signals, especially in older adults. Age-invariant relationships between neural selectivity at perception and retrieval have been demonstrated before (Johnson et al., 2015; Hill et al., 2021; Katsumi et al., 2021). In line with this research, our findings suggest that regardless of age, neural specificity during feedforward processing is correlated with neural specificity of feedback. We extend this postulation by demonstrating that this age-invariant relationship is only present for information retrieved episodically but not semantically and that it holds even in the absence of reduced neural differentiation of episodic feedback. The lack of relation between feedforward and semantic feedback aligns well with Park et al. (2010), who showed that neural specificity was associated with measures of fluid processing ability but not crystallized knowledge. A possible interpretation is that as accumulated knowledge increases, it is less dependent on the quality of neural differentiation in feedforward visual input. On the contrary, episodic memory ability is more variable across the lifespan, and the integrity of stored episodic content depends on the initial quality of neural differentiation at perception. Hence, episodic memory is more prone to differences in neural selectivity regardless of age.

In sum, we demonstrated the coexistence of concurrent contextual and time-distant mnemonic information in feedback signals in early visual areas V1 and V2 across age. Furthermore, we showed that individual feedback components follow distinct trajectories regarding neural dedifferentiation. Episodic feedback signals were comparably differentiated across age, whereas semantic feedback signals showed better neural differentiation in older adults than in younger adults, probably reflecting the lifelong accumulation of generalized knowledge. Notably, while feedforward differentiation was reduced in older adults, it was positively correlated with episodic feedback in both age groups. This suggests that measuring the dedifferentiation of internally generated signals depends on the nature of the retrieved information. Our findings have important implications for the investigation of memory reinstatement and aging, highlighting dissociations among different components of feedback signals across the lifespan.
